# Sub‐Ensemble Isolation in SERF Magnetometry Enabled by Micrometer‐Scale Polarization Control

**DOI:** 10.1002/nap2.70213

**Published:** 2026-07-20

**Authors:** Yuhao Zhang, Zihua Liang, Lu Liu, Jinsheng Hu, Peng Zhou, Gen Hu, Gaopu Hou, Mao Ye

**Affiliations:** ^1^ Key Laboratory of Ultra‐Weak Magnetic Field Measurement Technology Ministry of Education School of Instrumentation and Optoelectronic Engineering Beihang University Beijing China; ^2^ Zhejiang Provincial Key Laboratory of Ultra‐Weak Magnetic‐Field Space and Applied Technology Hangzhou Innovation Institute Beihang University Hangzhou China; ^3^ Hefei National Laboratory Hefei China

**Keywords:** space‐variant polarization metasurface, spin‐exchange relaxation‐free, sub‐ensemble isolation

## Abstract

Conventional understanding of a spin‐exchange relaxation‐free (SERF) atom ensemble pertains to the common perception that the rapid exchange of atom state finally results in uniform time evolution of the whole ensemble. However, in this study, we demonstrate that by manipulating pumping polarization in a micrometer level, misalignment between the time evolution of different sub‐ensembles can be created within a single SERF ensemble with unprecedented independency. A novel pumping system consisting of a miniaturized ^87^Rb vapor cell and a space‐variant polarization metasurface is developed for the proof of concept. Our method induces position‐dependent atomic anisotropy in both pumping and absorption into the thermal atomic ensemble. By constructing calculated Zeeman‐sublevel populations in the SERF regime, distinct sensing channels are generated with 0.22 V/nT average scale factor, which is comparable with a single channel generated by a single SERF ensemble. The average crosstalk ratios between adjacent channels are measured up to 32 dB, through the excitation of the fictitious magnetic field (3.5 nT, 30 Hz), which is measured as 20 dB without sub‐ensemble isolation in the same experimental condition. Our work demonstrates unprecedented crosstalk reduction in single‐cell multichannel SERF magnetometry, which holds new promise for applications including high‐spatial resolution neural biomagnetism mapping and portable magnetism measurement devices.

## Graphical abstract

Inter‐channel crosstalk caused by atomic diffusion fundamentally limits the spatial resolution of single‐cell multi‐channel SERF magnetometers. In this work, a spatial‐variant polarization metasurface is employed to achieve effective sub‐ensemble isolation within a single SERF atomic ensemble, which enables the suppression of inter‐channel crosstalk and demonstrates a promising route toward improved spatial resolution.

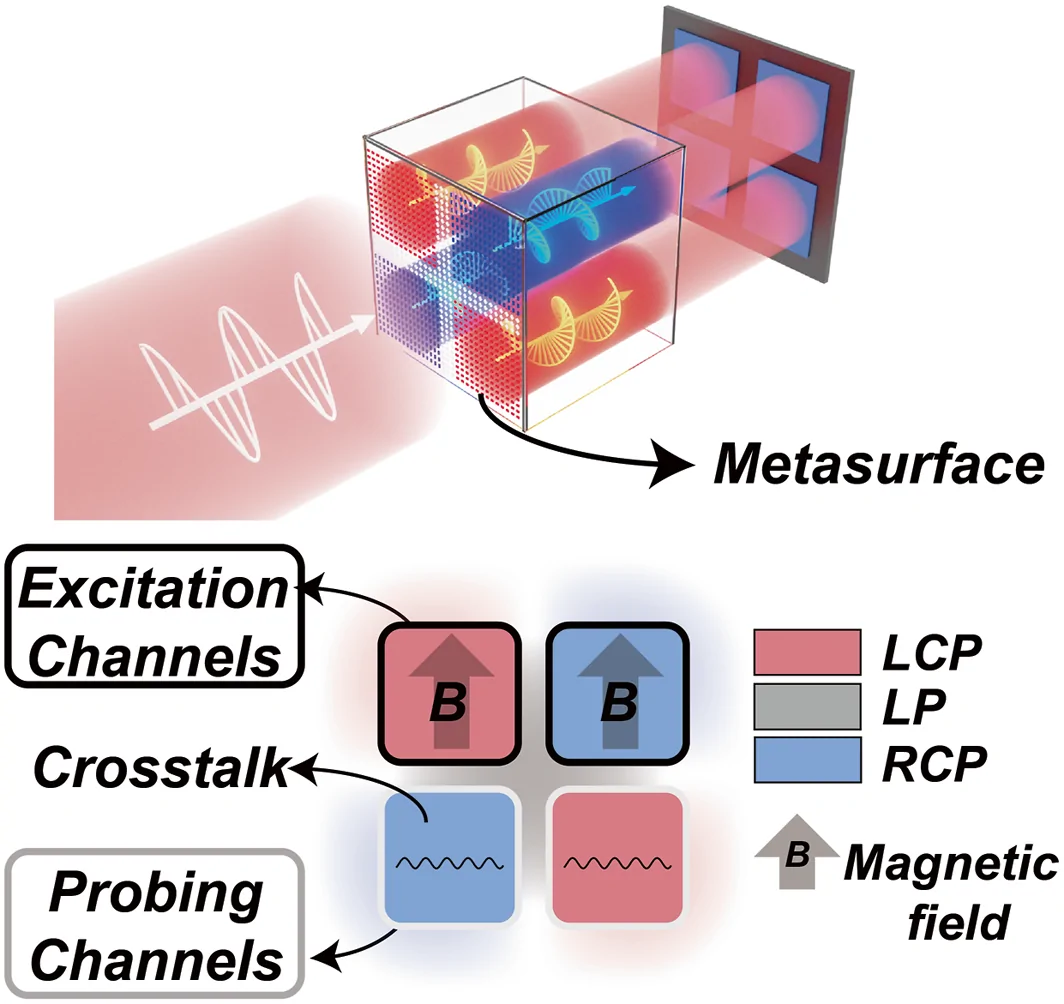

## 1 Introduction

The conventional understanding of spin‐exchange relaxation‐free (SERF) atomic ensembles pertains to the common perception that rapid spin‐exchange interactions lead to a uniform time evolution of the entire ensemble. This uniformity prolongs the spin relaxation time of alkali atoms, which is essential for ultra‐high‐sensitivity magnetic field measurements [1, 2]. However, this uniformity also limits an independent manipulation and interrogation of spin polarization, constraining the spatial resolution required for applications such as sub‐millimeter high‐resolution biomagnetism imaging [3, 4, 5]. Although additional reductions in size, weight, and power can be achieved by further scaling down the numerous individual components of atomic magnetometers [6], the existing spatial resolution of arrayed magnetometers remains limited to the centimeter scale by the trade‐off between sensitivity and probe size. [4, 7, 8, 9, 10, 11, 12]. Recent works have also explored hybrid optical pumping within a single vapor cell to improve the signal‐to‐noise ratio (SNR) [13, 14]. Despite DMD (digital micromirror device)‐ and AOM (acousto‐optic modulator)‐based dynamic light‐field modulation enabling virtual channel partitioning at the submillimeter scale [15, 16, 17], their inherent optoelectronic complexity fundamentally impedes chip‐scale integration and broader application in biomagnetism. In addition, emerging single‐cell multi‐channel magnetometers offer a promising approach to address the miniaturization and high‐resolution required to enable sub‐millimeter pixel imaging in biomagnetism measurement, but bring with them a new set of challenges in reducing magnetic field crosstalk between sensing channels caused by atomic diffusion [18, 19]. Recent studies demonstrate that inter‐channel crosstalk suppression can be achieved by physically separating detection channels beyond the atomic diffusion length [20, 21, 22, 23]. However, such approaches inherently involve critical drawbacks: sophisticated optical configurations are required, which result in a large system volume and signal attenuation caused by multi‐layer beam routing. These problems become particularly challenging when scaling to high‐density arrays in biomagnetism applications, as conventional isolation methods are not applicable to emerging microfabricated vapor cells or compatible with CMOS [24, 25].

Recent advancements in photonics and nanofabrication have led to the development of wavefront‐shaping methods based on subwavelength‐spaced arrays of optical elements (metasurfaces) [26, 27, 28, 29]. These methods have received widespread attention in integrated atomic magnetometers due to their versatile beam manipulation capabilities and scalability for compact device integration [30, 31, 32, 33]. Additionally, integrated photonics enables a chip‐scale integration of sophisticated functionalities, including the light delivery and collection processes required for atom interrogation in a SERF magnetometer [34, 35, 36, 37].

In this study, we demonstrate that the micrometer‐scale manipulation of the pumping polarization can induce controlled misalignment in the time evolution of distinct sub‐ensembles within a single SERF ensemble. By inducing position‐dependent atomic anisotropy in both pumping and absorption into the ^87^Rb thermal atomic ensemble, this polarization‐imposed channel isolation (PICI) pumping scheme creates spatially separated detection channels with antiparallel macroscopic spin polarization. Theoretical modeling reveals that the polarization gradient introduced by the PICI pumping scheme reduces the relaxation distance by 46% compared to the uniform pumping scheme based on a quarter‐wave plate (QWP), thereby suppressing inter‐channel crosstalk in single‐vapor‐cell multi‐channel magnetometers. For the proof of concept, a $2\times 2\;{\text{mm}}^{2}$ space‐variant polarization conversion metasurface is designed, fabricated, and finally characterized through Stokes measurement. Combining this metasurface with SERF magnetometry systems, a multichannel magnetometer system is established with an averaged scale factor of 0.22V/nT. To experimentally validate the inter‐channel crosstalk reduction of this system, a localized fictitious excitation method is introduced, enabling precise micron‐scale excitation of specific sub‐regions within the SERF ensemble. Crosstalk is quantified by comparing the response signal ratios of adjacent channels under identical local magnetic field excitation across the two pumping schemes. Compared with the conventional uniformly polarized pumping scheme that achieves an average crosstalk ratio of 20 dB, the PICI pumping scheme demonstrates an average crosstalk ratio of up to 32 dB between adjacent sensing channels. This 12 dB average enhancement corresponds to a 74.9% average crosstalk reduction, indicating the potential for improved spatial resolution. With this micrometer level pumping polarization, the PICI pumping scheme provides a promising glimpse into future compact, high‐sensitivity, and high‐resolution biomagnetism imaging and paves the way for advanced portable atomic sensing applications.

## 2 Results

### 2.1 Principle of the PICI Pumping Scheme

In atomic ensembles, the steady‐state spin‐polarization distribution is determined by the competition between homogenizing mechanisms, such as atomic thermal diffusion, and spatially nonuniform driving mechanisms, including optical pumping, wall‐collision boundary conditions, and magnetic‐field distributions. Consequently, the spin ensemble remains spatially nonuniform even in the SERF regime [38]. Based on this principle, the PICI pumping scheme is introduced to enhance the spatial nonuniformity of spin polarization, thereby forming distinguishable spin regions and suppressing crosstalk in a multichannel magnetometer.

In contrast to conventional single‐cell multi‐channel zero‐field SERF magnetometers, where optical polarization transformed by the bulky optics is controlled to uniformly transfer the same photon spin to the whole atomic ensemble, magnetometers based on the PICI pumping scheme utilize beams with structured polarization to manipulate photon spin point by point at the nanoscale across the transverse plane, thereby creating antiparallel macroscopic spin polarization and naturally separated sensing channels as shown in Figure 1a. This versatile polarization response is designed to generate space‐variant polarization when incident by linear polarized light: left‐/right‐handed circular polarizations in four distinct areas surrounded by linear polarizations along their edges—equivalent to sequential interactions with multiple polarizers and waveplates. These areas give rise to four spatially distinguishable sub‐ensembles within a single atomic ensemble corresponding to four sensing channels. Here, the term “sub‐ensemble” refers to spatially distinguishable portions of the atomic ensemble that exhibit different steady‐state spin polarizations under spatially varying optical pumping conditions.

**FIGURE 1:**
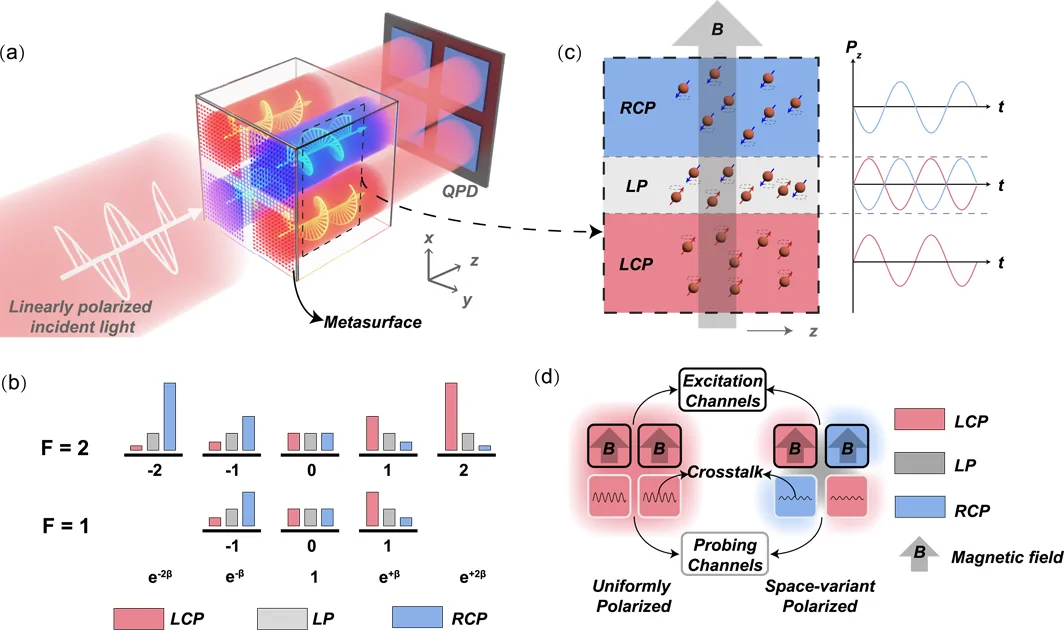
Concept of polarization‐imposed channel isolation (PICI). (a) Schematic of multichannel magnetometers with PICI. The spirals within the vapor cells indicate left‐/right‐handed circular polarization; the red and blue regions indicate electron spin with opposite directions. (b) Zeeman sublevel populations with different polarized pumping beams in the SERF regime, utilizing an alkali metal vapor cell filled with nitrogen gas. (c) Electron spin in the PICI pumping scheme. B, magnetic field; $P_{z}$, electron spin polarization. (d) Schematic of the crosstalk for the traditional uniformly polarized pumping scheme and the PICI pumping scheme. LCP, left‐handed circular polarization; LP, linear polarization; RCP, right‐handed circular polarization.

Pumped by the aforementioned space‐variant polarized beam, adjacent atomic sub‐ensembles are pumped into distinct Zeeman sublevels (as shown in Figure 1b) through photon spin transfer, creating antiparallel macroscopic spin polarization between adjacent circular polarized pumping channels as shown in Figure 1c [10, 39]. Influenced by the magnetic field B, the electron spins in these pumping channels generate regular precession signals $P_{z}$ with a 180‐degree relative phase difference. Moreover, due to the presence of equally distributed left‐ and right‐handed circular polarizations, the total electron spin $P_{z}$ in the LP (linear polarization) pumping region undergoes mutual cancellation, thereby making it insensitive to the variant in magnetic field. This inherent characteristic, leveraged through spatial separation and polarization gradients, enables an effective distinction of adjacent sensing channels and reduces signal crosstalk caused by thermal diffusion as shown in Figure 1d. Furthermore, higher‐order photon angular momentum modes in the PICI pumping scheme induce corresponding electron spin polarization modes. These polarization modes exhibit a faster relaxation process due to inhomogeneity in electron spin compared to fundamental modes [38, 40], thereby reducing relaxation distance and crosstalk between channels, which is detailed in Section 2.2 and the Supporting Information S1. In addition, due to the position‐dependent atomic absorption anisotropy, linearly polarized components are completely absorbed by the vapor cell, whereas circular polarized components remained, physically separating four sensing channels in the transmitted light as shown in Figure 1a [41].

To experimentally validate the inter‐crosstalk reduction of the PICI pumping scheme over conventional uniformly polarized light pumping, a localized fictitious magnetic field is used to excite specific regions as shown in Figure 1d. Influenced by the located magnetic field as shown as the black arrows, signals with the same frequency and different amplitudes can be detected in adjacent sensing channels (represented by boxes with white borders in Figure 1d). Due to lower electron spin between channels and higher‐order photon angular momentum mode, pumping beam with space‐variant polarization exhibits lower crosstalk compared with conventional uniformly polarized pumping scheme.

### 2.2 Theoretical Analysis: Relaxation Due to Polarization Inhomogeneity

The time evolution of spin polarization *P* in the alkali metal atomic ensemble, simultaneously influenced by optical pumping, relaxation, and Larmor precession, can be phenomenologically described by the Bloch equation [41]:

(1) $$\begin{array}{c} \frac{\partial }{\partial t}P=\frac{\gamma_{e}}{Q}B\times P+\frac{1}{Q}\left[R_{\text{op}}\left(s\hat{z}-P\right)-R_{\text{rel}}P\right]\\ +D\nabla^{2}P \end{array}$$

B is the magnetic field, $\gamma_{e}$ is the electron gyromagnetic ratio, Q is the nuclear slowing‐down factor, D is the diffusion coefficient, and $R_{\text{op}}$ and $R_{\text{rel}}$ are the optical pumping rate and relaxation rate, respectively. Although conventional theoretical models predominantly assume spatially homogeneous spin polarization distributions within atomic vapor cells, this work expands the electronic spin polarization P(r) and optical angular momentum field s(r) using the eigenstates $u_{nm}\left(r\right)$ of the Laplacian operator $\nabla^{2}$ to investigate the relaxation dynamics mediated by the excitation of higher‐order atomic polarization modes through photonic angular momentum modulation [38].

(2) $$P\left(r\right)=\sum_{n,m}P^{\left(nm\right)}u_{nm}\left(r\right)$$

(3) $$s\left(r\right)=\sum_{n,m}s^{\left(nm\right)}u_{nm}\left(r\right)$$

(4) $$P^{\left(nm\right)}=\int_{\Omega }P\left(r\right)u_{nm}^{*}\left(r\right)dr$$

(5) $$s^{\left(nm\right)}=\int_{\Omega }s\left(r\right)u_{nm}^{*}\left(r\right)dr$$

where Ω represents the two‐dimensional region corresponding to the cross‐section of the atomic vapor cell. By substituting Equations ((2), (3), (4), (5)) into Equation (1), the time involution of each spatial mode coefficient is obtained under Dirichlet boundary conditions as follows:

(6) $$\begin{array}{c} P_{+}^{\left(nm\right)}\left(t\right)=-i\frac{\gamma_{e}}{Q\left(i\omega_{q}-1/T_{nm}\right)}B_{+}P_{z}^{\left(nm\right)}\left(1-e^{i\omega_{q}t-t/T_{nm}}\right) \end{array}$$

where $T_{nm}$ denotes the transverse relaxation time of the nth‐order mode, given by

(7) $$\frac{1}{T_{nm}}=\frac{R_{\text{op}}+R_{\text{rel}}}{Q}+Dk_{nm}^{2}.$$

$k_{nm}$ is the eigenvalue of the Laplacian operator corresponding to the order (*n*,*m*) mode. As the mode order increases, $k_{nm}$ becomes larger, leading to shorter transverse relaxation times. Since the diffusion length scales as $\sqrt{DT_{nm}}$, the sub‐ensemble polarization exhibits weaker diffusion capability for higher‐order modes.

Equation (6) describes the temporal evolution of the transverse polarization modes of each order. The time‐varying term comprises a rotating oscillatory term at the Larmor frequency $\left(\omega_{q}\right)$ and an exponential decay term with the relaxation time of each order as the decay constant. Expressing the overall transverse polarization as a sum of the individual modes, we obtain

(8) $$P_{+}\left(t,r\right)=\sum_{n,m}P_{+}^{\left(nm\right)}\left(t\right)\;u_{nm}\left(r\right)$$

Define the effective relaxation time $t_{0}$ as the time required for the total error to decay to 1/e of its maximum value:

(9) $$\begin{array}{c} \sum_{n,m}{\left|\frac{\gamma_{e}B_{+}}{Q\left(i\omega_{q}-1/T_{nm}\right)}\right|}^{2}{\left|P_{z}^{\left(nm\right)}\right|}^{2}e^{-2t_{0}/T_{nm}}\\ =e^{-2}\sum_{n,m}{\left|\frac{\gamma_{e}B_{+}}{Q\left(i\omega_{q}-1/T_{nm}\right)}\right|}^{2}{\left|P_{z}^{\left(nm\right)}\right|}^{2}. \end{array}$$

According to Equation (8), the temporal evolution of the polarization distribution can be expressed as a weighted superposition of individual modes. Therefore, for different configurations that generate distinct polarization distributions, those with a larger contribution from higher‐order modes impose a stronger restriction on the diffusion of the sub‐ensemble polarization. We analyze the differences in the spatial distribution modes of the polarization generated by the conventional uniform and PICI pumping scheme based on their distinct symmetry properties. The corresponding effective transverse relaxation times are calculated to be $t_{1}=4.58\;ms$ and $t_{2}=2.31\;ms$, respectively. Detailed derivations and analysis can be found in Supporting Information S1.

This theoretical analysis indicates that, within a simplified model, the PICI pumping scheme exhibits shorter relaxation time compared to the conventional uniform scheme, which may suppress diffusion and inter‐channel crosstalk. Because direct resolution of spatial differences between uniform and nonuniform polarization across multiple channels is experimentally challenging, localized excitation and the measurement of crosstalk in other channels are used as an indirect validation in the experimental section.

### 2.3 Design, Fabrication, and Characterization of the Metasurface

Significant challenges in multi‐parameter optimization must be overcome to accurately achieve polarization control in this PICI pumping scheme. In particular, a large‐scale parametric dataset must be constructed through a systematic variation of critical dimensions, followed by rigorous filtering to identify geometric configurations that satisfy the orthogonally aligned principal axes condition. Implementing this methodology demands substantial computational resources and often results in limited design accuracy. Inverse‐design and machine‐learning techniques will be key tools for addressing this challenge by searching nonintuitive design spaces.

In this study, a reverse optimization algorithm is developed to directly optimize the structural dimensions of the nano‐fin unit by using the DOP (degree of polarization) as the fitness function [33, 34]. The detailed design methodology and process of nanofabrication are provided in the Supporting Information S1.

The meta‐atoms of the three polarization transformers are schematically illustrated in Figure 2a. The optical micrographs of the space‐variant polarization conversion metasurface in Figure 2b express very characteristic patterns. The SEM image shown in Figure 2c reveals the interface of LP/RCP regions and the details of the meta‐atoms in a linear polarization region. Subsequently, a Stokes parameter measurement system, consisting of a quarter‐wave plate (QWP), linear polarizer, and beam imaging system, is employed to systematically characterize the performance of this space‐variant polarizer [42]. The experimental results displayed in Figure 2d demonstrate that the transmitted light achieves a clear space‐variant polarization distribution. In addition, the fabricated metasurface exhibits a transmission efficiency of approximately 73.2%, which is lower than that of a standard QWP and may reduce the signal‐to‐noise ratio to some extent. Nevertheless, conventional isolation methods generally rely on multiple additional optical components that also introduce optical losses [20, 21]. By enabling sub‐ensemble isolation through a space‐variant polarization distribution without requiring extra polarization‐control optics, the proposed metasurface may simplify the optical architecture and maintain competitive system‐level optical efficiency.

**FIGURE 2:**
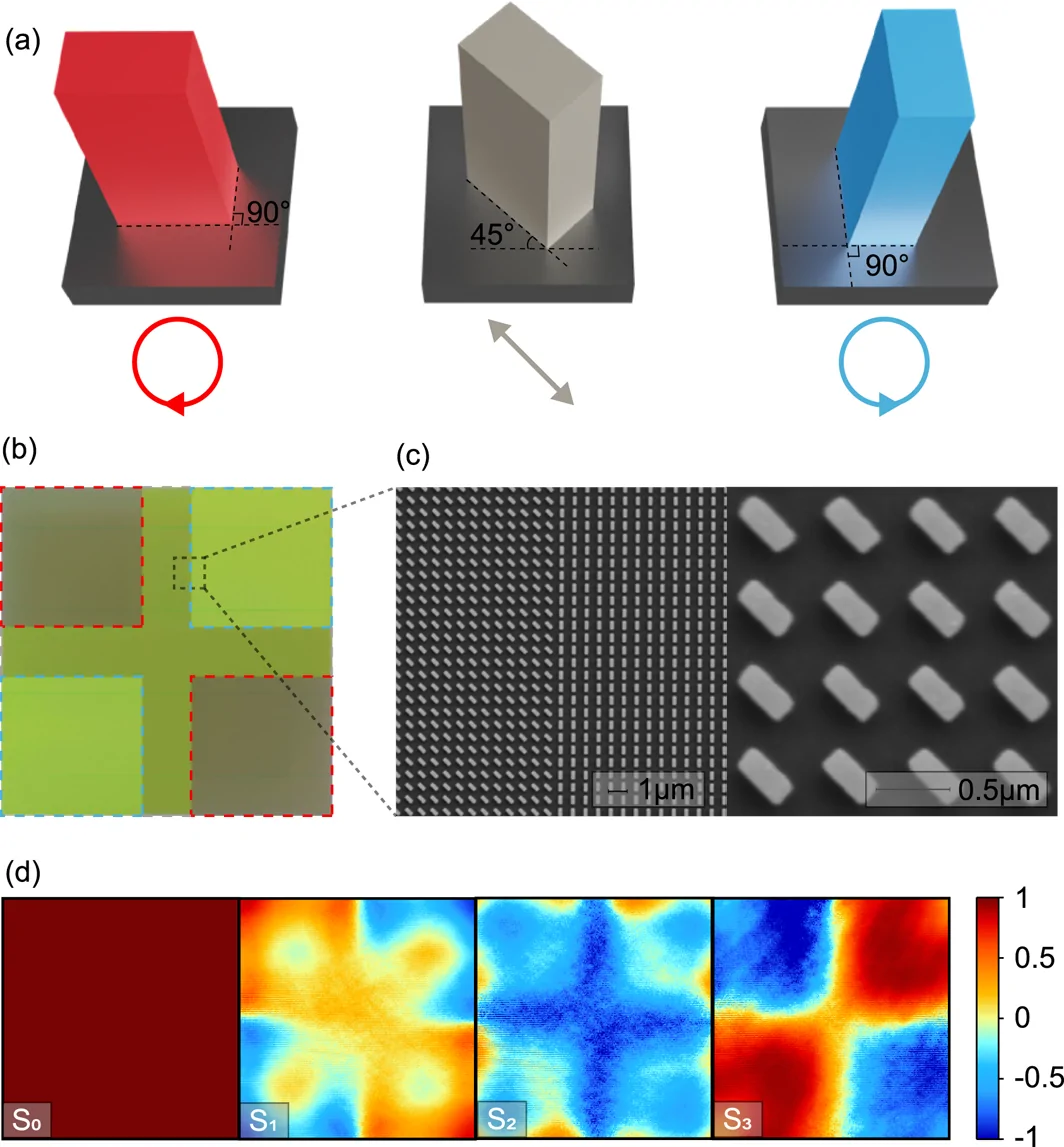
Design, characterization, and testing of the space‐variant polarization conversion metasurface. (a) Diagram of the meta‐atoms for three polarization transformers; (b) optical micrographs and (c) SEM images of the space‐variant polarization conversion metasurface; (d) measured Stokes parameters of the space‐variant polarization conversion metasurface.

### 2.4 Crosstalk Characterization via Located Fictitious Magnetic Field

For the comparison of crosstalk between the proposed PICI pumping scheme and the conventional QWP‐based scheme, it is critical to accurately excite a single channel with a spatially restricted magnetic field and then monitor the crosstalk in the adjacent channels. However, the current coil‐based magnetic field control systems in conventional magnetometers are ill‐equipped to solve this challenge. Therefore, this research leverages a localized fictitious magnetic field to selectively excite specific sub‐regions in the atomic ensemble as shown in Figure 3a and compares the amplitude ratio (Channel A/Channel C) between the two schemes based on the metasurface and QWP. After passing through the vapor cell as shown in Figure 3a, the intensity of the pumping beam is simultaneously detected by four independent channel photodetectors (quadrant photodiode detector, QPD), forming a four‐channel atomic magnetometer sensitive to the spatial distribution of the magnetic field. At the same time, a detuned circularly polarized beam is incident on the vapor in the direction perpendicular to the pumped light, covering the pumped light as completely as possible. Subsequently, the controlled fictitious magnetic field is generated inside the vapor cell by modulating the intensity of the detuned circularly polarized light using an electro‐optical modulator (EOM). Influenced by optical modulation, which is divided into multiple test intervals, a response signal proportional to the fictitious magnetic field is captured by the photodetector. In addition, a movable optical mask is utilized to limit the range of the modulated light, thereby generating a spatially controlled fictitious magnetic field within the vapor cell. However, limited by a millimeter spot size and isolated test environments, it is still very challenging to precisely control the fictitious magnetic field so that it only affects channel AB while detecting crosstalk on channel CD as shown in Figure 3a. Thus, the entire displacement process of the optically masked, from fully masked to fully unmasked modulated light, is broken down into multiple test intervals. The ratio of signal amplitudes between Channels A and C is recorded in each mask position as an indirect method of assessing crosstalk between the channels of the two pump schemes. The experimental setup is shown in Figure 3b and the specific setup and steps are detailed in Section 4.

**FIGURE 3:**
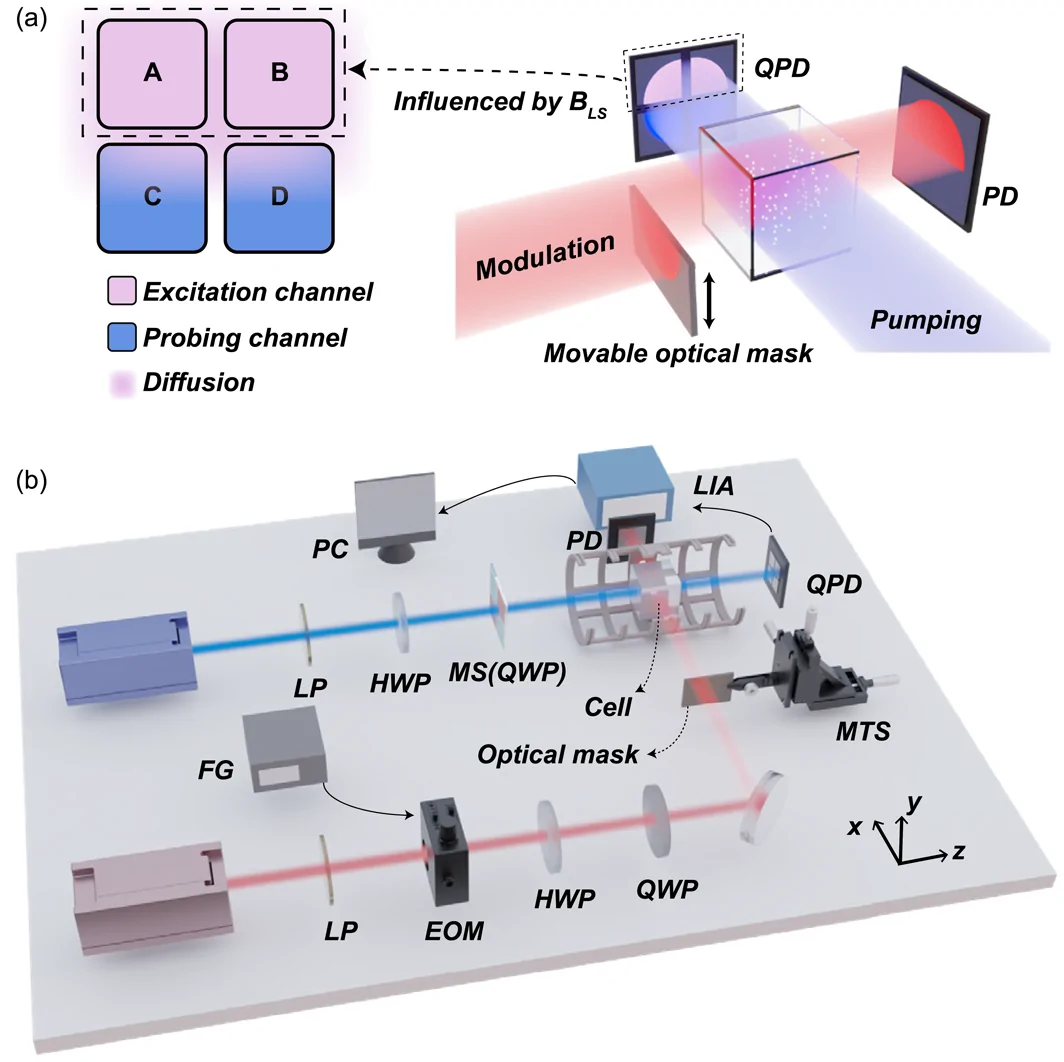
Experimental setup for crosstalk characterization through optically induced fictitious magnetic field. (a) Diagram of the localized excitation method: By modulating the incident light (red) and spatially confining it with an optical mask, a measurable fictitious magnetic field is generated within a localized region of the pump light (blue); (b) experimental setup for the characterization of crosstalk. EOM, electro‐optic modulator; FG, function generator; LIA, lock‐in amplifier; MS, metasurface; MTS, manual linear translation stages; QPD, quadrant photodiode (with 100 μm channel spacing).

Define the position‐dependent isolation performance (PDIP) as

(10) $$PDIP\left(x\right)=20\log_{10}\left(\frac{\left|V_{A}\left(x\right)\right|}{|V_{C}\left(x\right)|Q_{c}}\right)$$

where $V_{A}\left(x\right)$ and $V_{C}\left(x\right)$ represent the complex amplitudes of the 30‐Hz response of the main Channel A and the adjacent Channel C at position x (obtained by Fourier transform). $Q_{c}$ denotes the signal conversion efficiency of adjacent Channel C (in order to eliminate the inherent errors between channels due to detector sensitivity, magnetometer scale factor, etc.). In addition, the systematic error in the PDIP measurement may arise from optical alignment errors, photodetector‐induced systematic error, and positioning error of manual linear translation stages (MTS).

### 2.5 Experimental Results

Based on the experimental setup, a comparative evaluation of the magnetometer response and crosstalk suppression for the metasurface‐based PICI pumping scheme and the conventional QWP‐based pumping scheme is shown in Figure 4. By scanning the magnetic field $B_{x}$ in the SERF regime, the dispersion‐like magnetic response curves and scale factors of channels A (0.2 V/nT) and C (0.24 V/nT) are demonstrated in Figure 4a. The discrepancy in scale factors likely originates from nonuniform light intensity distribution and variations in photoelectric conversion efficiency among different areas of QPD. To address these systematic variations, signal conversion efficiency $Q_{c}$ is introduced into the calculation of the PDIP. This correction ensures accurate crosstalk quantification independent of inherent sensor responsivity differences. Figure 4b tracks the amplitude responses of Channels A and C as the optical mask translates from full occlusion (*x* = 0) to complete exposure (*x* = 2.4 mm). Due to inherent alignment limitations in both the pumping scheme, which makes it challenging to precisely operate the optical mask to fully occlude Channel C while completely exposing Channel A, the entire displacement range is evenly discretized into 13 test intervals with 200 μm increments. For example, Figure 4c indicates the time‐domain amplitude response curves of the exciting channel and probing channel under uniform circularly polarized pumping with the optical mask positioned at *x* = 1.8 mm. Figure 4d shows the equivalent noise power spectral density (ENPSD) of channel A under the two pumping schemes. Around 30Hz, the noise floor reaches approximately $0.2\;pT/Hz^{1/2}$. The PDIP at this position is calculated from the 30‐Hz components (corresponding to the modulation frequency of the fictitious magnetic field), where

(11) $${\text{PDIP}}_{\text{QWP}}\left(1.8\right)=20\log_{10}\left(\frac{\left|V_{A}\left(1.8\right)\right|}{|V_{C}\left(1.8\right)|Q_{C}}\right)=25.9\;\text{dB}$$

**FIGURE 4:**
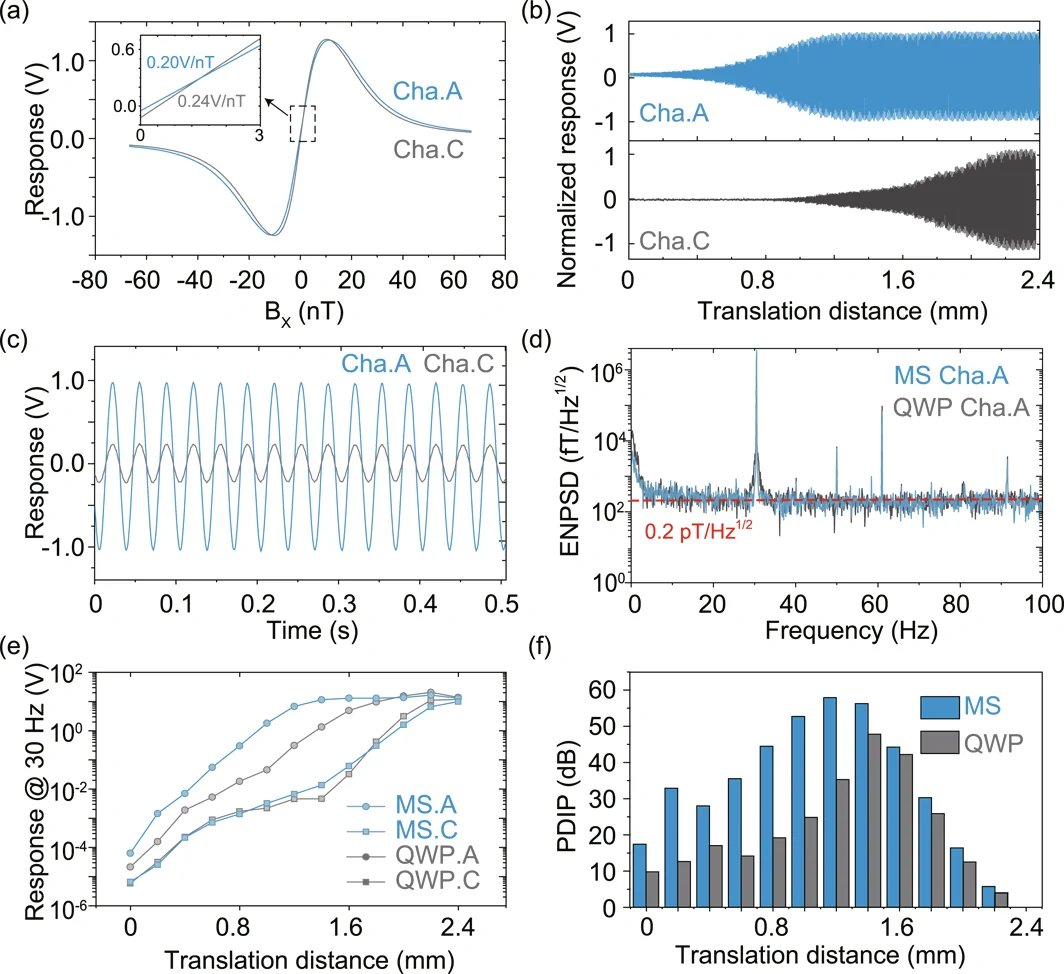
Experimental results of the single‐cell multi‐channel magnetometer. (a) Magnetic response curves and scale factors of Channels A and C; (b) amplitude responses of Channels A and C during continuous optical mask translation (0 ∼ 2.4 mm range); (c) time‐domain amplitude responses of primary/adjacent channels under the uniform pumping scheme (optical mask position: x=1.8mm); (d) the equivalent noise power spectral density (ENPSD) of Cha. A under two pumping schemes; (e) amplitudes of 30 Hz signals (channels A/C) under two pumping schemes in response to the location of optical mask; (f) position‐dependent isolation performance (PDIP, channels A/C) under the PICI scheme and the uniform pumping scheme.

This value quantifies the amplitude ratio between channels, serving as an evaluation for crosstalk in different pumping schemes. Following this methodology, Figure 4e indicates the responses of the 30‐Hz responses for Channels A and C under schemes based on the space‐variant polarization conversion metasurface and QWP. Instead of directly inferring crosstalk intensity from the absolute signal magnitudes in Channel A or Channel C, we quantified crosstalk strength by calculating the ratio of signal amplitudes between Channel A and Channel C (A/C ratio) at each stage of the magnetic field's influence. This approach provides a robust indirect method for assessing crosstalk between the channels for the two pump schemes. As shown in Figure 4f, the metasurface (MS)‐based experimental system demonstrates a higher A/C response ratio compared to the quarter‐wave plate (QWP) method, indicating superior crosstalk suppression capability. Notably, the larger signal magnitude observed in Channel A for the MS method in Figure 4e reflects the higher response efficiency of the MS system under Channel A excitation, rather than an amplification of crosstalk. The A/C ratio analysis, corroborated by the data in Figure 4f, clearly highlights the MS method's advantage in minimizing crosstalk. Notably, in the PICI pumping scheme, signals in Channel C exhibit a pronounced temporal delay relative to those in Channel A, probably due to the establishment of distinct spatial boundaries caused by linearly polarized light absorption mechanisms.

Furthermore, repeated experiments show that although the exposure area progressively increases with optical mask displacement, the response amplitude in each channel is not strictly monotonically increasing. Peaks of amplitude exceeding adjacent position are likely attributed to mask‐induced optical diffraction. Figure 4f compares channel isolation performance between pumping schemes. It is observed that the metasurface‐based pumping scheme suppresses more crosstalk at each mask location. Compared with the conventional uniformly polarized pumping scheme that achieves a 20‐dB average crosstalk ratio, the PICI pumping scheme demonstrates up to 32 dB average crosstalk ratio between adjacent sensing channels. This 12 dB average enhancement corresponds to a 74.9% average crosstalk reduction, indicating the potential for improved spatial resolution. In addition, the difference between the PDIPs of the two pumping schemes reaches a maximum of 27.9 dB at x=1mm, corresponding to a 94.7% relative crosstalk reduction. These results are directly attributable to spatial optical polarization isolation enabled by the space‐variant polarization conversion metasurface, which induces isolation of atomic spin polarization in a single SERF ensemble.

## 3 Discussion

Spin‐exchange relaxation‐free (SERF) atomic ensembles are traditionally understood to exhibit uniform time evolution due to rapid spin‐exchange interactions that homogenize atomic states across the ensemble. In this study, we show that the micrometer‐scale modulation of the pumping polarization enables controlled divergence in the dynamics of spatially distinct sub‐ensembles within a single SERF system, offering a new degree of independence previously unattainable. By combining the space‐variant polarization metasurface with a thermal atomic ensemble in the SERF regime, antiphase spin polarization is imposed on the atomic ensemble by the construction of calculated Zeeman sublevel populations, leading to the separation of distinct sensing channels. This result corresponds to a 74.9% average crosstalk reduction, indicating the potential for improved spatial resolution. However, the increased spin‐exchange relaxation rate between channels, which is essential for effective channel separation, may impact the magnetometer's sensitivity. Sensitivity and spatial resolution are critical performance metrics for SERF magnetometers, and practical applications often necessitate a careful trade‐off between these parameters. The key advantage of the proposed method lies in its flexibility, which enables tailored optimization of system parameters to meet specific application requirements. Thus, this method provides a viable solution for achieving high spatial resolution in single‐cell multi‐channel magnetometers while offering a versatile framework for optimizing performance across diverse application scenarios. However, as the array density increases, the LP zones will inevitably occupy a larger fraction of the sensing area, resulting in increased effective dead space and reduced signal intensity. Therefore, in practical implementations, the channel size and LP‐zone width should be carefully optimized to balance signal strength and crosstalk suppression. Future work will explore integration with VCSELs [43, 44] and micro‐fabricated vapor cells toward chip‐scale SERF magnetometers.

## 4 Materials and Methods

A $8\times 8\times 8\;{\text{mm}}^{3}$ borosilicate cubic cell containing a droplet of ^87^ Rb and 720Torr of $N_{2}$ buffer gas serves as a sensitive source for magnetometers, heated to 150°C using a twisted pair winding resistor driven by a 240‐kHz AC electronic current [43]. A multilayer cylindrical magnetic shield composed of four layers of μ‐metal and an aluminum shield achieves a quasistatic shielding factor of at least $10^{5}$, guaranteeing a low‐noise magnetic environment with a residual magnetic field measuring less than 5 nT. The outermost layer of aluminum shielding is designed to reduce high‐frequency magnetic noise. Furthermore, a cylindrical uniform field coil is employed to control the triaxial magnetic field inside the cylindrical magnetic shield [45, 46, 47]. A collimated 795‐nm circularly polarized beam (diameter: 2.3 mm, power: 650 μW) propagating orthogonally to the primary pump laser (D1 line, $\sigma^{+}$, 1 mW) is frequency‐detuned by +80 GHz from the Rb D1 transition to minimize absorption while maintaining sufficient light shift. The beam intensity is sinusoidally modulated at 30 Hz using an electro‐optic modulator (Thorlabs, NEL03A/M), generating a time‐varying fictitious magnetic field described by

(12) $$B_{LS}\left(t\right)=B_{0}\;\sin\left(60\pi_{m}t\right),\;B_{0}=3.5\;\text{nT}.$$

An optical mask on a manual linear stage (LBTEK, LS65‐13) is utilized to spatially control the overlapping volume between the excitation and pumping beams, thereby confining the fictitious magnetic field into targeted submillimeter zones. The optical mask, translated vertically in 200 μm increments, spatially tunes the influence of the fictitious magnetic field—progressively shifting from no channel coverage to isolated activation of Channel A (at about 50% exposure) and finally to full‐channel coverage (Channels A and C at 100% transmittance).

The detailed experimental procedure is as follows:

1. Initialize the magnetometer in the spin‐exchange relaxation‐free (SERF) regime [41] (*T* = 150°C, ^87^ Rb density $n_{\text{Rb}}=1.01\times 10^{14}{\text{cm}}^{-3}$).
2. Set the optical mask to start position (*x* = 0 mm, no channel interference) and acquire 60 s noise floor data.
3. Perform stepwise translation (13 positions, *x* = 0 mm to *x* = 2.4 mm) with 200 μm steps.
4. At each position, apply the modulated fictitious field $\left(B_{LS}\left(t\right)=B_{0}\;\sin\;60\pi_{m}t\right)$; simultaneously measure the 30‐Hz signal amplitudes in the stimulated channel (A) and adjacent channel (C) using a synchronized dual‐phase lock‐in amplifier (Zurich Instruments MFLI), enabling crosstalk quantification at the modulation frequency of the fictitious magnetic field.

## Author Contributions

**Yuhao Zhang:** methodology, formal analysis, software, data curation, validation, writing – original draft, visualization, investigation. **Zihua Liang:** conceptualization, methodology, software, data curation, validation, investigation, writing – original draft, visualization. **Lu Liu:** conceptualization, methodology, validation, software, visualization. **Jinsheng Hu:** validation, methodology, visualization, investigation. **Peng Zhou:** methodology, validation, investigation. **Gen Hu:** validation, methodology. **Gaopu Hou:** validation, methodology. **Mao Ye:** conceptualization, writing – review and editing, supervision, project administration, resources, funding acquisition.

## Funding

This work was supported by the National Natural Science Foundation of China for Excellent Young Scholars (Overseas) (Grant No. 37110101), the National Natural Science Foundation of China (Grant No. 77051001), and the Innovation Program for Quantum Science and Technology (Grant No. 2021ZD0300503).

## Ethics Statement

The authors have nothing to report.

## Consent

Informed consent was obtained from all individuals included in this study.

## Conflicts of Interest

The authors declare no conflicts of interest.

## Supporting information


Supporting Information S1

## Data Availability

The simulated and experimental data that support this study are available from the corresponding author upon reasonable request.
