# Supplementary material for: Sub‐Ensemble Isolation in SERF Magnetometry Enabled by Micrometer‐Scale Polarization Control
Source: Nanophotonics. 2026 Jul 20;15(14):e70213. doi: 10.1002/nap2.70213 (PMC13642543; doi:10.1002/nap2.70213)
Supplement: Supplementary file 1 [file NAP2-15-e70213-s001.pdf]

Research Article

Yuhao Zhang†, Zihua Liang†, Lu Liu, Jinsheng Hu, Peng Zhou, Gen Hu, Gaopu Hou, and Mao Ye*

Supplementary Material

1 The effect of photon angular momentum field on atomic sub-ensemble diffusion length

For a multi-channel magnetometer in a single vapor cell, the influence between channels, i.e.,inter-channel crosstalk, originates from the thermal diffusion of polarized atoms in a sub-ensemble carrying the magnetic field information of one channel, which interferes with that of another channel. In our previous work, we proposed a simple diffusion–convolution model to evaluate the influence of different spatial distributions of the photon angular momentum field on polarization diffusion under the simplified condition of an infinite one-dimensional system. The results show that non-uniform distributions can reduce the diffusion length, thereby partially suppressing inter-channel crosstalk[1]. However, the previous model is based on overly idealized and simplified assumptions. In the following, we employ a mode analysis approach to investigate the effect of different angular momentum distributions on the diffusion length of the sub-ensemble polarization in the two-dimensional transverse plane within the vapor cell.

The temporal evolution of spin polarization $\boldsymbol{P}$ in the alkali metal atomic ensemble can be phenomenologically described by the Bloch equation [2]:

$$\frac{\partial}{\partial t}\boldsymbol{P} = \frac{\gamma}{Q}\boldsymbol{B}\times\boldsymbol{P} + \frac{1}{Q}[R_{\mathrm{op}}(s\hat{z}-\boldsymbol{P}) - R_{\mathrm{rel}}\boldsymbol{P}] + D\nabla^2\boldsymbol{P} \tag{1}$$

Besides temporal evolution, $\boldsymbol{P}$ also exhibits a spatial distribution due to atomic thermal diffusion and wall collisions.

Assuming that the spin polarization drops to zero upon wall collisions, the polarization distribution $\boldsymbol{P}(\boldsymbol{r})$ satisfies the homogeneous Dirichlet boundary condition $\boldsymbol{P}(\boldsymbol{r}) = 0$ at the vapor-cell walls. We expand $\boldsymbol{P}(\boldsymbol{r})$ in terms of the eigenstates $u_{nm}(\boldsymbol{r})$ of the Laplacian ∇^2 as

$$\boldsymbol{P}(\boldsymbol{r}) = \sum_{n,m}\boldsymbol{P}^{(nm)}u_{nm}(\boldsymbol{r}) \tag{2}$$

where $u_i(\boldsymbol{r})$ satisfy the homogeneous Dirichlet boundary conditions. Since the magnetic field is measured only within the vapor cell, the photon angular momentum distribution field $s_0(\boldsymbol{r})$ can be

0 † These authors contributed equally.

Yuhao Zhang†, Zihua Liang†, Lu Liu, Jinsheng Hu, Peng Zhou, Gen Hu, Gaopu Hou, Key Laboratory of Ultra-Weak Magnetic Field Measurement Technology, Ministry of Education, School of Instrumentation and Optoelectronic Engineering, Beihang University, Beijing 100191, China; Zhejiang Provincial Key Laboratory of Ultra-Weak Magnetic-Field Space and Applied Technology, Hangzhou Innovation Institute, Beihang University, Hangzhou 310051, China; Hefei National Laboratory, Hefei 230088, China

***Corresponding author: Mao Ye,** Key Laboratory of Ultra-Weak Magnetic Field Measurement Technology, Ministry of Education, School of Instrumentation and Optoelectronic Engineering, Beihang University, Beijing 100191, China; Zhejiang Provincial Key Laboratory of Ultra-Weak Magnetic-Field Space and Applied Technology, Hangzhou Innovation Institute, Beihang University, Hangzhou 310051, China; Hangzhou Institute of Extremely-Weak Magnetic Field Major National Science and Technology Infrastructure, Hangzhou 310051, China; Hefei National Laboratory, Hefei 230088, China

written as

$$s(\boldsymbol{r}) = h(\boldsymbol{r}) s_0(\boldsymbol{r}) \tag{3}$$

$h(\boldsymbol{r})$ is a window function, defined as $h(\boldsymbol{r}) = 1$ for $\boldsymbol{r}$ inside the vapor cell, and $h(\boldsymbol{r}) = 0$ otherwise.

$$h(\boldsymbol{r}) = \begin{cases} 1, (\boldsymbol{r}) \in \Omega^\circ \\ 0, (\boldsymbol{r}) \notin \Omega^\circ \end{cases} \tag{4}$$

where Ω represents the two-dimensional region corresponding to the cross-section of the atomic vapor cell.

$s(\boldsymbol{r})$ can also be expanded as

$$s(\boldsymbol{r}) = \sum_{n,m} s^{(nm)} u_{nm}(\boldsymbol{r}) \tag{5}$$

The coefficients $s^{(nm)}$ and $\boldsymbol{P}^{(nm)}$ are given by the overlap integrals

$$\boldsymbol{P}^{(nm)} = \int_\Omega \boldsymbol{P}^{(nm)}(\boldsymbol{r}) u^*_{nm}(\boldsymbol{r}) d\boldsymbol{r} \tag{6}$$

$$s^{(nm)} = \int_\Omega s(\boldsymbol{r}) u^*_{nm}(\boldsymbol{r}) d\boldsymbol{r} \tag{7}$$

Substituting Eq.5 7 into Eq.1 yields

$$\begin{aligned} \frac{d}{dt} \sum_{n,m} \boldsymbol{P}^{(nm)} u_{nm}(\boldsymbol{r}) = \frac{\gamma_e}{Q} \boldsymbol{B} \times \sum_{n,m} \boldsymbol{P}^{(nm)} u_{nm}(\boldsymbol{r}) - \frac{R_{\text{tot}}}{Q} \sum_{n,m} \boldsymbol{P}^{(nm)} u_{nm}(\boldsymbol{r}) + \\ \frac{R_{\text{op}}}{Q} \sum_{n,m} s^{(nm)} u_{nm}(\boldsymbol{r}) \hat{z} + D \sum_{n,m} \boldsymbol{P}^{(nm)} \nabla^2 u_{nm}(\boldsymbol{r}) \end{aligned} \tag{8}$$

where $R_{tot} = R_{op} + R_{rel}$. Rewriting the above equation using the eigenvalue of the ∇^2, we obtain

$$\begin{aligned} \frac{d}{dt} \sum_{n,m} \boldsymbol{P}^{(nm)} u_{nm}(\boldsymbol{r}) = \frac{\gamma_e}{Q} \boldsymbol{B} \times \sum_{n,m} \boldsymbol{P}^{(nm)} u_{nm}(\boldsymbol{r}) - \frac{R_{\text{tot}}}{Q} \sum_{n,m} \boldsymbol{P}^{(nm)} u_{nm}(\boldsymbol{r}) + \\ \frac{R_{\text{op}}}{Q} \sum_{n,m} s^{(nm)} u_{nm}(\boldsymbol{r}) \hat{z} - D \sum_{n,m} \boldsymbol{P}^{(nm)} k^2_{nm} u_{nm}(\boldsymbol{r}) \end{aligned} \tag{9}$$

As derived from the equation above, if the angular momentum distribution of light s is considered to have spatial variation while the intensity (corresponding to R_{op}) remains spatially uniform, different modes will exhibit no coherence with each other and evolve independently[3]. So, the Bloch equation of each order is

$$\frac{d}{dt} \boldsymbol{P}^{(nm)} = \frac{\gamma_e}{Q} \boldsymbol{B} \times \boldsymbol{P}^{(nm)} - \frac{R_{\text{tot}}}{Q} \boldsymbol{P}^{(nm)} + \frac{R_{\text{op}}}{Q} s^{(nm)} \hat{z} - D \boldsymbol{P}^{(nm)} k^2_{nm} \tag{10}$$

Let $B_+ = B_x + iB_y, P_+ = P_x + iP_y$. Decomposing the equation into transverse (x, y) and longitudinal (z) components, where the z-component is assumed to reach a steady-state value driven by optical pumping along the z-axis that generates macroscopic spin polarization, results in the following: the magnetic field components in the x and y directions produce weak Larmor precession, which is negligible relative to the dominant effect of the optical pumping process, yields

$$-\frac{R_{\text{tot}}}{Q} P_z^{(nm)} + \frac{R_{\text{op}}}{Q} s^{(nm)} - D P_z^{(nm)} k^2_{nm} = 0 \tag{11}$$

$$\frac{d}{dt} P_+^{(nm)} = i \left(P_+^{(nm)} \omega_q - \frac{\gamma_e}{Q} P_z^{(nm)} B_+ \right) - \frac{R_{\text{tot}}}{Q} P_+^{(nm)} - D P_+^{(nm)} k^2_{nm} \tag{12}$$

where $\omega_q = \gamma_e B_z/Q$ denote the Larmor precession frequency. Solving the above equation yields the steady-state values of the longitudinal (z-direction) atomic polarization modes at each order

$$P_z^{(nm)} = \frac{R_{\text{op}} T_{nm} s^{(nm)}}{Q} \tag{13}$$

where T_{nm} represents the relaxation time for each order of polarization modes

$$\frac{1}{T_{nm}} = \frac{R_{\text{tot}}}{Q} + Dk_{nm}^2. \tag{14}$$

Substituting the transverse polarization modes' evolution equations into the system and simplifying yields

$$\frac{d}{dt}P_+^{(nm)} = (i\omega_q - \frac{1}{T_{nm}})P_+^{(nm)} - i\frac{\gamma_e}{Q}B_+ P_z^{(nm)} \tag{15}$$

The solution to this differential equation is

$$P_+^{(nm)}(t) = -i\frac{\gamma_e}{Q(i\omega_q - 1/T_{nm})}B_+ P_z^{(nm)}\left(1 - e^{i\omega_q t - t/T_{nm}}\right) \tag{16}$$

For a square vapor cell satisfying Dirichlet boundary conditions, the eigenfunctions $u_{nm}(\boldsymbol{r})$ in Cartesian coordinates are

$$u_{nm}(x,y) = A\sin\frac{n\pi}{a}(x+\frac{a}{2})\sin\frac{m\pi}{a}(y+\frac{a}{2}) \tag{17}$$

for $-\frac{a}{2} \le x \le \frac{a}{2}$ and $-\frac{a}{2} \le y \le \frac{a}{2}$ with the eigenvalue k_{nm}

$$k_{nm}^2 = k_n^2 + k_m^2 = (\frac{n\pi}{a})^2 + (\frac{m\pi}{a})^2 \tag{18}$$

where A is the normalization constant, a is the side length of the square vapor cell, and is taken to be $a = 3\,\text{mm}$ in the following simulations. n and m denote the mode indices in the x- and y-directions, respectively. It is evident that the symmetry of the eigenfunctions is determined by the indices n and m: when n (m) is odd, the eigenfunction is even with respect to the y-axis (x-axis), whereas when n (m) is even, it is odd with respect to the corresponding axis. Fig. S2 shows the results of theoretical calculations of steady-state distribution and transient response of transverse polarization for the $3\times3\times3$ mm^3 vapor cell with typical parameters $D = 0.18\text{cm}^2/\text{s}$, $R_{\text{op}} = 400\text{s}^{-1}$, $R_{\text{rel}} = 400\text{s}^{-1}$ and $T = 150°\text{C}$. Based on the equation derived above, the relaxation times of the $(1,1)$ and $(2,2)$ modes of $s(\boldsymbol{r})$ are calculated to be $T_1 = 3.46\,\mu\text{s}$ and $T_2 = 1.87\,\mu\text{s}$, respectively.

Eq.16 describes the temporal evolution of the transverse polarization modes of each order. The time-varying term comprises a rotating oscillatory term at the Larmor frequency (ω_q) and an exponential decay term with the relaxation time of each order as the decay constant. Expressing the overall transverse polarization as a sum of the individual modes, we obtain:

$$P_+(t,\boldsymbol{r}) = \sum_{n,m} P_+^{(nm)}(t)\,u_{nm}(\boldsymbol{r}) \tag{19}$$

The steady-state temporal error is:

$$err(t,\boldsymbol{r}) = -ie^{i\omega_q t}\sum_{n,m}\frac{\gamma_e}{Q(i\omega_q - 1/T_{nm})}B_+ P_z^{(nm)} e^{-t/T_{nm}} u_{nm}(\boldsymbol{r}) \tag{20}$$

Integrating the magnitude of $err(t,\boldsymbol{r})$ over space and using the orthogonality of the eigenfunctions $u_{nm}(\boldsymbol{r})$, the total error becomes:

$$|err|^2 = \int_\Omega err(t,\boldsymbol{r})\,err^*(t,\boldsymbol{r})\,d\boldsymbol{r} = \sum_{n,m}\left|\frac{\gamma_e B_+}{Q(i\omega_q - 1/T_{nm})}\right|^2 \left|P_z^{(nm)}\right|^2 e^{-2t/T_{nm}} \tag{21}$$

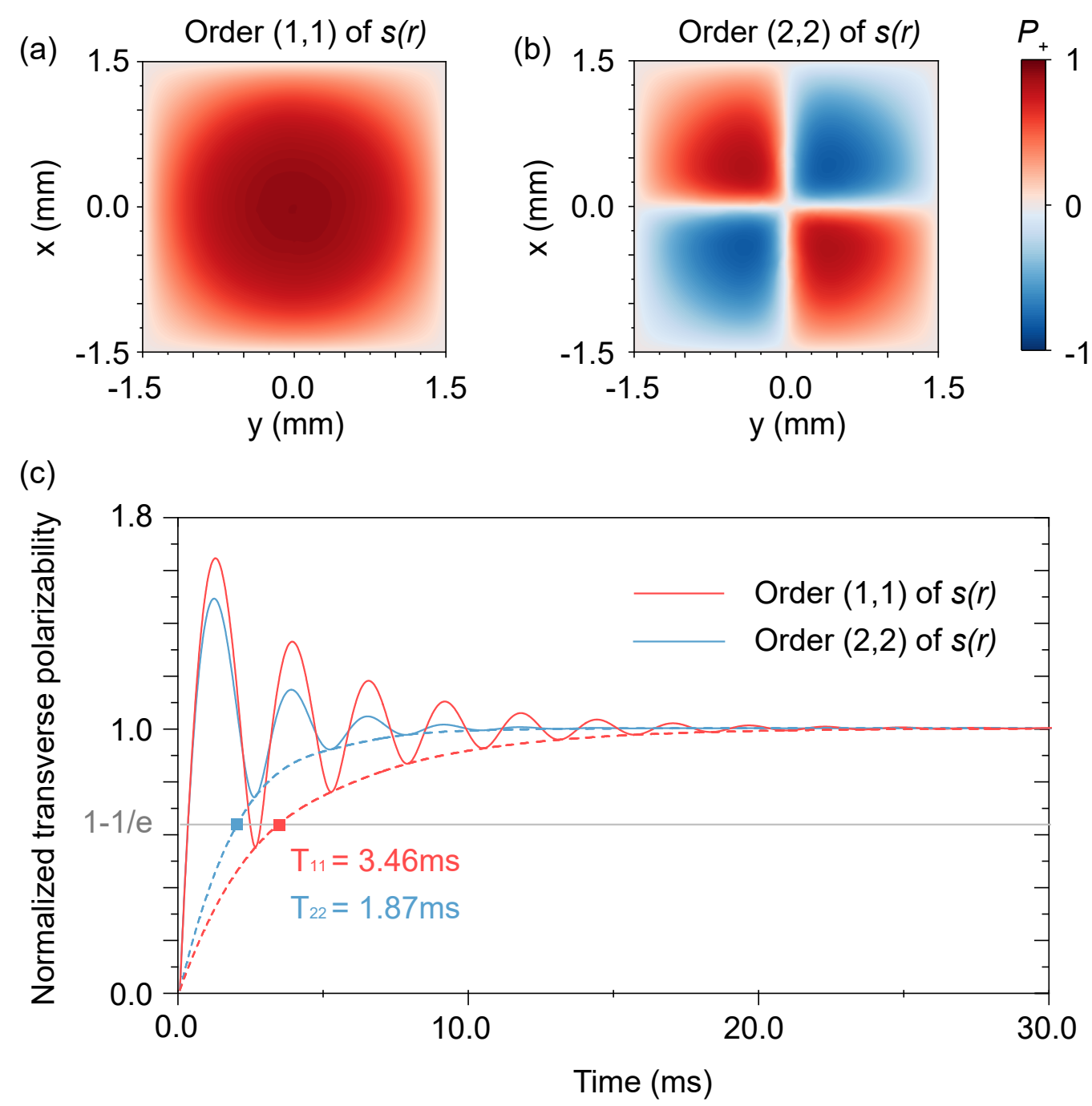

Figure S1: Steady-state distribution and transient response of transverse polarization. Fundamental mode of $P_+(t)$ induced by (a) (0, 0) and (b) (1, 1) mode of $s(\boldsymbol{r})$; (c) The transient response of overall transverse polarization induced by $(1,1)$ and (b) $(2,2)$ order distribution of photon angular momentum.

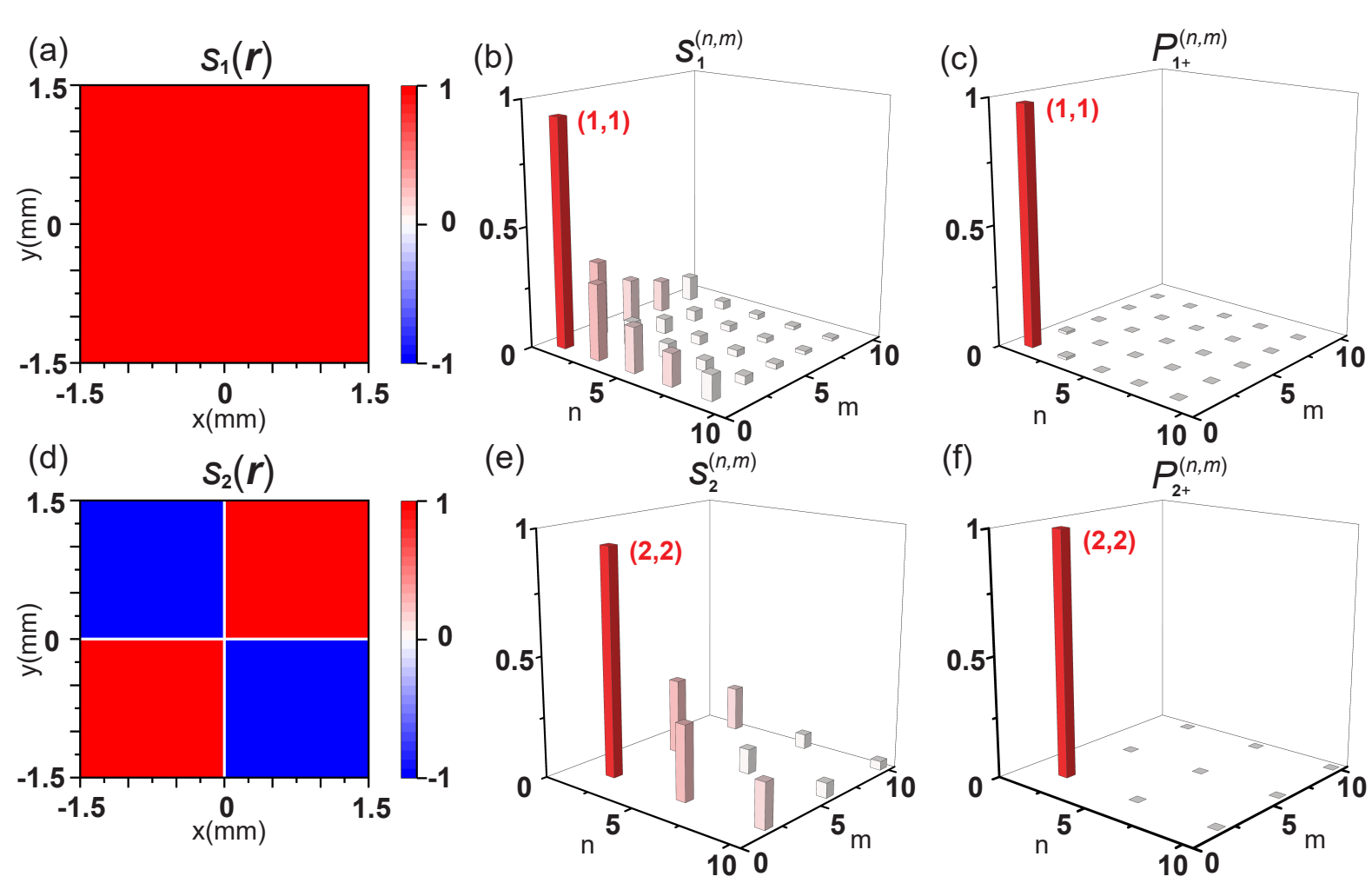

Figure S2: Calculated mode components under different photon angular momentum fields.(a) and (d) are the distribution of two different photon angular momentum fields; (b) and (e) are normalized mode coefficients of two fields; (c) and (f) are the normalized mode coefficients of polarization distribution. The modes excited by these two fields are predominantly of low order; however, the dominant modes excited by the second field are of higher order than those excited by the first field.

Define the effective relaxation time t_0 as the time required for the total error decays to $1/e$ of its maximum value:

$$\sum_{n,m}\left|\frac{\gamma_e B_+}{Q(i\omega_q-1/T_{nm})}\right|^2\left|P_z^{(nm)}\right|^2 e^{-2t_0/T_{nm}}=e^{-2}\sum_{n,m}\left|\frac{\gamma_e B_+}{Q(i\omega_q-1/T_{nm})}\right|^2\left|P_z^{(nm)}\right|^2. \tag{22}$$

Based on the above derivation, the temporal evolution of the polarization is linear for each spatial mode, and the overall polarization can be expressed as a weighted superposition of all mode components. Different orders of P_n correspond to different transverse relaxation times T_{nm}, which decrease with increasing mode order according to Eq.14. Since the diffusion length scales as $\sqrt{DT_{nm}}$, higher-order modes exhibit shorter diffusion lengths. Therefore, employing an angular momentum distribution that excites higher-order spatial modes of the polarization can effectively suppress diffusion between the corresponding sub-ensembles and consequently reduces inter-channel crosstalk between channels.

In the experiment, we employed two angular momentum distributions, as shown in Fig.S2(a) and (d). For the first, uniform distribution, it exhibits even symmetry with respect to both axes, and thus only odd-order modes $(2n-1, 2m-1)$ which exhibit even symmetry with $n, m = 1, 2, \ldots$, are present. In contrast, the second distribution exhibits odd symmetry with respect to both axes, and therefore only even-order modes $(2n, 2m)$, with $n, m = 1, 2, \ldots$, are present. This selection rule arises from the orthogonality of eigenfunctions with different parity. Considering that the dominant mode components are of relatively low order, and that Eqs.13 and 16 establish a one-to-one correspondence between the angular-momentum mode coefficients $s^{(nm)}$ and the transverse polarization mode coefficients $P_+^{(nm)}$, the second distribution is expected to excite higher-order polarization modes, resulting in shorter diffusion lengths for the corresponding sub-ensembles. According to Eq.20, the effective transverse relaxation times for the two distributions are $t_1 = 4.58\,\mathrm{ms}$ and $t_2 = 2.31\,\mathrm{ms}$, respectively, with the corresponding diffusion lengths satisfying $l_1 > l_2$. Under the first (uniform) distribution, atoms in the sub-ensemble encoding the magnetic-field information of one channel are more likely to diffuse into the measurement region of another sub-ensemble, leading to stronger inter-channel crosstalk compared with the second distribution.

2 Design and fabrication of the metasurface

2.1 Inverse Design Method

The algorithm utilized in this study incorporates the particle swarm algorithm and is comprised of five principal steps[4, 5].

- Step 0: Algorithm parameter initialization: Structure parameters (p, w, l, h, α) that associated with the SOP of the transmission response are set as variables with limited range in the algorithm. The initial set of nanopillar particles is generated through the random assignment of values to each particle parameter.
- Step 1: Evaluate SOP by Finite Difference Time Domain (FDTD) calculation: The software was employed to calculate the SOP of the nanopillar transmission response, defined as the ratio of the short-axis length to the long-axis length, and to return the current parameter set and SOP to the algorithm for further calculations.
- Step 2: Update the optimal value of the optimization parameter: The current parameter set is compared with the historical optimum, and if the latter value is exceeded, the current parameters are substituted with the historical optimum.
- Step 3: Update speed and location of particles: The parameter set is modified based on the current position and velocity of each particle.

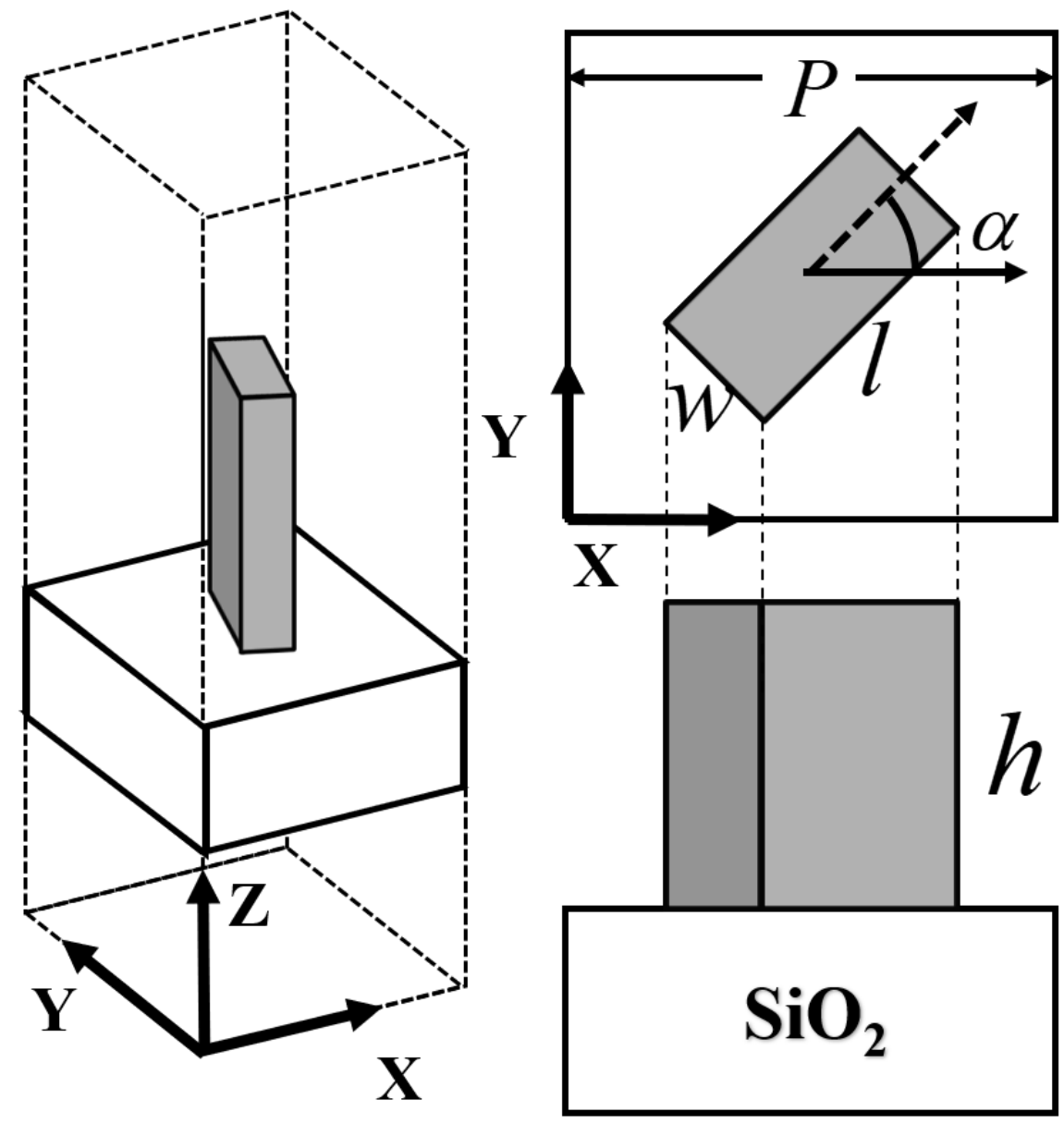

Figure S3: Schematic of the metasurface composed of the nanopillars.

– Step 4: Evaluate termination conditions: Check if the ellipticity meets the criteria or if the number of iterations reaches the limit. If so, record the historical optimal value and parameter group; otherwise, start the next iteration.

2.2 Process of Nanofabrication

First, a 550 nm a-Si film was deposited on a fused silica substrate through plasma enhanced chemical vapour deposition (PECVD). Next, a layer of 220 nm resist ZEP 520A was spin-coated on top for electron-beam lithography (EBL), which was performed using JEOL 6300FS at an acceleration voltage of 100 kV with a 225 $\mu C/cm^2$ dose. After EBL and development, the patterned sample was coated with 15 nm Cr_2O_3using evaporation. The deposition rate was 6 nm/min at room temperature and a background vacuum of 4×10^{-6} $Torr$. A lift-off process was then used to form the pattern of the Cr_2O_3 hard mask by soaking the sample in N-methyl-2-pyrrolidinone (PG remover from Kayaku Advance Materials). To transfer the pattern from the mask to the a-Si layer, an inductively coupled plasma reactive ion etching (ICP-RIE) process was carried out with a mixture of C_4F_8 and $SF_6$to etch all uncovered a-Si area. Finally, the sample was soaked in the Cr Etchant (Transene Company, Inc.) to remove the remaining Cr_2O_3. The preferential use of chromium oxide Cr_2O_3 over metallic chromium Cr as an etch mask stems from its inherently reduced lateral etching and superior structural fidelity. This oxide-mediated suppression of sidewall erosion arises from fluorine-induced surface passivation during plasma etching, enabling near-ideal anisotropic profiles critical for nanoscale metasurface patterning.

References

[1] Z. Liang, Y. Zhang, T. Long, B. Li, J. Hu, P. Zhou, L. Liu, G. Hu, M. Ye, and Y. Wang, "Four-channel miniaturized serf magnetometer with spatial inhomogeneous atomic polarization," *Optics & Laser Technology*, vol. 181, p. 112013, 2025.

[2] S. J. Seltzer, *Developments in alkali-metal atomic magnetometry*. Princeton University, 2008.

[3] W. Wang, M. Xia, W. Quan, and K. Wei, “Mode analysis of spin field of thermal atomic ensembles,” *Quantum Science and Technology*, vol. 9, no. 3, p. 035048, 2024.
[4] Z. Liang, J. Hu, P. Zhou, L. Liu, G. Hu, A. Wang, and M. Ye, “Metasurface-integrated elliptically polarized laser-pumped serf magnetometers,” *Microsystems & Nanoengineering*, vol. 10, no. 1, p. 101, 2024.
[5] Z. Liang, T. Long, L. Liu, J. Hu, P. Zhou, G. Hu, Y. Wang, and M. Ye, “Space-variant polarization distribution mitigates ac-stark shifts in a serf atomic magnetometer,” *ACS Photonics*, vol. 12, no. 5, pp. 2522–2529, 2025.
